# Supplementary material for: Association of expenditure on ultra-processed foods and beverages and anthropometric indicators in Mexican children: A longitudinal study
Source: PLoS One. 2025 Mar 11;20(3):e0317831. doi: 10.1371/journal.pone.0317831 (PMC11896057; doi:10.1371/journal.pone.0317831)
Supplement: Attachment — Submitted filename: Response to Reviewers.docx [file pone.0317831.s004.docx]

**Response to reviewers**

Reviewers' comments:

Reviewer's Responses to Questions

Comments to the Author

1. Is the manuscript technically sound, and do the data support the conclusions?

The manuscript must describe a technically sound piece of scientific research with data that supports the conclusions. Experiments must have been conducted rigorously, with appropriate controls, replication, and sample sizes. The conclusions must be drawn appropriately based on the data presented.

Reviewer #1: Yes

Reviewer #2: No

**Response: Thank you for the comment. The conclusions in lines 367-371 have been revised to ensure consistency with the analyzed data and the results obtained. Although the study does not involve experiments, it was applied a rigorous methodology in conducting this observational study, as detailed in (a) Van Belle G, Fisher L, Heagerty P, Lumley T. Longitudinal Data Analysis. In: Biostatistics: A Methodology for the Health Sciences. In Wiley-Interscience; 2004. p. 728–65, (b) Verbeke, G. & Molenberghs, G. Linear mixed models for longitudinal data. (Springer Science & Business Media, 2000), (c) Liang KY, Zeger SL. Longitudinal Data Analysis Using Generalized Linear Models. Biometrika. 1986;73(1):13-22. These references were also included in the original manuscript.**

2. Has the statistical analysis been performed appropriately and rigorously?

Reviewer #1: Yes

Reviewer #2: I Don't Know

**Response: The statistical analysis was conducted appropriately and rigorously. Descriptive analysis was performed using standard methodology. As referred to in the manuscript, association analysis was carried out using two generally accepted methods (random-effects linear regression models and generalized estimating equation models), their assumptions were verified, details of their specifications were provided, and their results were thoroughly compared.**

3. Have the authors made all data underlying the findings in their manuscript fully available?

The [PLOS Data policy](http://c05y1x9s.r.us-east-2.awstrack.me/L0/http:%2F%2Fwww.plosone.org%2Fstatic%2Fpolicies.action%23sharing/1/010f019295e51fa3-f4cf4698-b905-4ed2-8fbb-70f4dae8f00c-000000/_sUWadBeqJ9kBUXECzGifdSAq_8=181) requires authors to make all data underlying the findings described in their manuscript fully available without restriction, with rare exception (please refer to the Data Availability Statement in the manuscript PDF file). The data should be provided as part of the manuscript or its supporting information, or deposited to a public repository. For example, in addition to summary statistics, the data points behind means, medians and variance measures should be available. If there are restrictions on publicly sharing data—e.g. participant privacy or use of data from a third party—those must be specified.

Reviewer #1: Yes

Reviewer #2: Yes

**Response: Indeed, all the information used in the study is publicly accessible, and the sources for consultation have been provided.**

4. Is the manuscript presented in an intelligible fashion and written in standard English?

PLOS ONE does not copyedit accepted manuscripts, so the language in submitted articles must be clear, correct, and unambiguous. Any typographical or grammatical errors should be corrected at revision, so please note any specific errors here.

Reviewer #1: Yes

Reviewer #2: No

**Response: Thank you for the observation. The original writing of the manuscript in English was supported by a certified translator. However, the document was reviewed again, with special emphasis on the abstract.**

5. Review Comments to the Author

Please use the space provided to explain your answers to the questions above. You may also include additional comments for the author, including concerns about dual publication, research ethics, or publication ethics. (Please upload your review as an attachment if it exceeds 20,000 characters)

Reviewer #1: The study explored the longitudinal association between expenditure on ultra-processed foods and beverages (UPF) and changes in anthropometric indicators of obesity with a representative sample. The study was well designed and the statistical analyses were applied appropriately. In the discussion section, I suggest including more studies that investigated the relationship between UPF consumption and obesity and other indicators of body adiposity than those that evaluated variables that were not the subject of their research, such as lipid profile. Below are some references that I suggest the authors consult to improve the discussion.

References
Neri, D., et al, Obesity reviews, 2021. 23 Suppl 1, e13387.
Canella DS et al. PLoS One. 2014 Mar 25;9(3):e92752
Silva FM et al. Public Health Nutr. 2018 Aug;21(12):2271-2279
Juul F et al. Br J Nutr. 2018 Jul;120(1):90-100
Monteiro CA et al. Public Health Nutr. 2018 Jan;21(1):18-26

**Response: We greatly appreciate the references provided by Reviewer 1 to enrich the discussion. We removed the paragraph that mentioned the studies with lipid profile as an outcome (lines 278-285) and we added several of the suggested references throughout the rest of the discussion.**

Reviewer #2: Abstract
background of the abstract is too long. Variables must be mentioned in method section. P values must be added to the results. It is better to write the duration instead of round 2.

**Response: Thank you for the suggestions. We shortened the background in the abstract, specified the exposure and outcome variables in the methods, added the p-value and the confidence interval in the results, and replaced the mention of rounds with the follow-up period.**
Introduction
Introduction is too long and more similar to a literature review. As there are lots of articles on children, it is not logical to mention the works on adults in the introduction. potential mechanisms/justifications for the relation of UPFs intake and obesity must be written. It is more suitable to condense the last paragraph in 2 sentences.

**Response: Thank you for the observation. We shortened the introduction, briefly mentioning the studies conducted in adults. We also mentioned the potential mechanisms for the relation between UPF intake and obesity. We condensed the final paragraph as suggested.**
Methods
please name the study's covariates. Add a reference for calculating the wealth index. Table of UPFs is not compete and lacks a lot of food items. 2 main determinants of weigh status are energy intake and physical activity, without measuring them the results could not be reliable.

**Response: Thank you for your suggestions. The study covariates were named in the "Covariates" subsection in a manner consistent with the names appearing in Table 1, which outlines the characteristics of the study sample, as well as with the rest of the document. A reference for the calculation of the wealth index has been added. In Fig. 2, it has been clarified that the classified foods and beverages are those with reported expenditure in the MxFLS. We acknowledge that energy intake and physical activity are two main determinants of weight status; therefore, we transformed the expenditure into an adult-equivalent measure that accounts for differences by age and gender and incorporated the variable of the proportion of time reported watching television to mitigate these potential biases, respectively. While the limitations already acknowledged the issue related to the use of adult-equivalent per capita expenditure, we added a mention of the lack of a direct measurement of physical activity.**
